# The use of inertial sensors system for human motion analysis

**DOI:** 10.1179/1743288X11Y.0000000006

**Published:** 2010-12

**Authors:** Antonio I Cuesta-Vargas, Alejandro Galán-Mercant, Jonathan M Williams

**Affiliations:** 1 Faculty of Health Sciences, University of Malaga, Spain; 2 Department of Life Sciences, Roehampton University, Whitelands College, London, UK

**Keywords:** Inertial sensors, Motion analysis, Review

## Abstract

**Objective:**

The aim of this article is to review systematically and appraise critically the literature surrounding the research, comparing inertial sensors with any kind of gold standard; this gold standard has to be a tool for measuring human movement (e.g. electrogoniometry, optoelectronic systems, electromagnetic systems, etc.).

**Method:**

A MEDLINE, EMBASE, CINAHL, PEDRo and SCOPUS search of published English language articles was conducted, which focused on articles that compared inertial sensors to any kind of gold standard (e.g. electrogoniometry, optoelectronic systems, electromagnetic systems, etc.), from 2000 to 2010. Two independent reviewers completed the study selection, quality appraisal and data extraction. The Critical Appraisal Skills Programme Español tool was used to assess study quality, and a reliability comparison between the systems was made.

**Results:**

Fourteen out of 242 articles were reviewed, which displayed a similar threat to validity, relating to sample selection and operator blinding. Other study limitations are discussed. A comparison between the different systems showed good agreement across a range of tasks and anatomical regions.

**Conclusions:**

This review concludes that inertial sensors can offer an accurate and reliable method to study human motion, but the degree of accuracy and reliability is site and task specific.

## Introduction

Kinematic measurements are used widely by clinicians and researchers alike. Such measures have been used to quantify both normal and pathological movements, quantify the degree of impairment, plan rehabilitation strategies and assess the effect of various interventions.

Clinical systems of motion analysis are often quick and simple to use; however, such systems often lack valuable kinematic data. Tape measures and goniometers provide information in single planes and only for static positions. Electrogoniometers and inclinometers may offer solutions for more than one plane, as well as provide dynamic data; however, the physical design of such sensors can restrict motion. Therefore, it remains difficult for the clinician to gain information about dynamic three-dimensional movements.

In contrast, laboratory systems are complex and expensive but are capable of resolving three-dimensional movements. Two laboratory systems commonly found within the literature are electromagnetic systems and video-based optoelectronic systems.

Electromagnetic tracking devices consist of a source that emits an electromagnetic field, which is used to determine the location and orientation of sensors. Such a system has been shown to be highly reliable and accurate.^1^–^14^ A limitation of electromagnetic systems is that they can be affected adversely by the presence of metals,^14^ the correction of which is lengthy and complicated,^15^ and accuracy can be compromised if the subject moves towards the edge of the defined operating field. This constrains the task that can be analyzed.

Video based optoelectronic systems are often thought of as the laboratory gold standard. This system utilizes retro-reflective markers visualized by multiple video cameras; such a set-up offers great flexibility, enabling the visualization of multiple body regions. It is possible to track motions in three-dimensions; however, inherent limitations include its complexity and time-consuming operation.^16^ Such a system is constrained by the operating environment due to line-of-sight difficulties, which can result in missed data.^5^

It is therefore clear that a large discrepancy exists between the currently available clinical systems of motion analysis and those used in the laboratory. Recently, new technology borrowed from aerospace, industrial and robotic engineering, appears a promising development in the field of motion analysis. Small, low-powered electromechanical sensors using technologies such as accelerometers, magnetometers and gyroscopes may be able to bridge the gap between large laboratory systems and clinical systems, providing the potential for dynamic three-dimensional motion analysis without the constraints outlined above. Numerous studies have reported using systems based on different types of inertial sensors, including (but not limited to) those based on accelerometers^17^–^21^ or gyroscopes;^15^,^22^–^24^ however, commonly, these two types of sensors (accelerometers and gyroscopes) are combined for the study of human motions, resulting in increased accuracy.^5^–^7^,^9^–^11^,^13^,^25^–^36^,^37^ Due to their small size and portability, these sensors could be an attractive option for ‘in the field’ motion analysis. However, before such technology can be used routinely, reliability and validity needs to be reviewed to compare its performance against a gold standard. Previous reviews have either focused on discussing the advantages and disadvantages of a variety of motion analysis systems^38^ or have provided a discussion of possible clinical applications.^16^

It is therefore the aim of this article to review systematically and appraise critically the literature surrounding the research comparing inertial sensors with accepted laboratory gold standards for measuring human movement (electrogoniometry, optoelectronic systems, electromagnetic systems, etc.). Such information would enable clinicians and researchers to determine whether this technology could be applied to a particular application.

## Methods

### Study identification and selection

The literature search began with the retrieval of published reports indexed on health-, biomechanics- and engineering-related electronic databases from MEDLINE, EMBASE, CINAHL, PEDro and SCOPUS. The search was performed to identify all possible studies pertinent to the research question. Search terms included validation, kinematic, inertial tracking devices and inertial sensor. The search was limited to the English language, humans and the last 10 years. We identified papers and relevant conference proceedings, which were hand searched. To be included in this review, studies had to meet the criteria outlined below (based on the PICO model).

- Population
  Including reports of the validation for a variety of body sensing regions.
- Intervention
  Validation and suitability of inertial sensors for human motion analysis.
- Control/comparison
  Accepted methods of human movement analysis (e.g. electrogoniometry, optoelectronic systems, electromagnetic systems, etc.).
- Outcomes
  Reliability coefficients and/or a measurement of error.

Studies were excluded if they did not meet the above criteria or if they dealt with drugs or surgery.

The review was conducted to examine its primary relevance to the measurement of human posture and movement. The title and abstracts identified by the initial search strategy were screened by the first named author to identify potentially eligible reports and retrieve full-text articles. When the title or abstract did not clearly indicate whether an article should be included, the complete article was obtained and reviewed.

### Type of study

Studies directly comparing inertial sensors with accepted methods of human motion analysis (e.g. electrogoniometry, optoelectronic systems, electromagnetic systems, etc.) formed the basis of this review.

### Data extraction

Data extraction was independently completed by two reviewers, with a consensus opinion adopted to resolve disagreement. A standardized data extraction tool was constructed to identify and detail key features of each study. The reviewers independently piloted the form with a small subset of representative studies to confirm its content. The extracted study details focused on participants’ characteristics, the anatomical region involved, study procedures, type of sensor/portability, the gold standard, biomechanical models and statistical analysis. Primary outcomes of accuracy and/or reliability were also extracted.

### Assessment of study quality

Two independent reviewers completed the quality appraisal, with disagreements resolved by consensus. The studies were critically appraised using the Critical Appraisal Skills Programme Español (CASPe) tool.^39^ Appraisal criteria were not applied to the conference proceedings or abstract-only reports because their brevity limited the provision of methodological detail.

## Results

### Study selection

**Figure 1:**
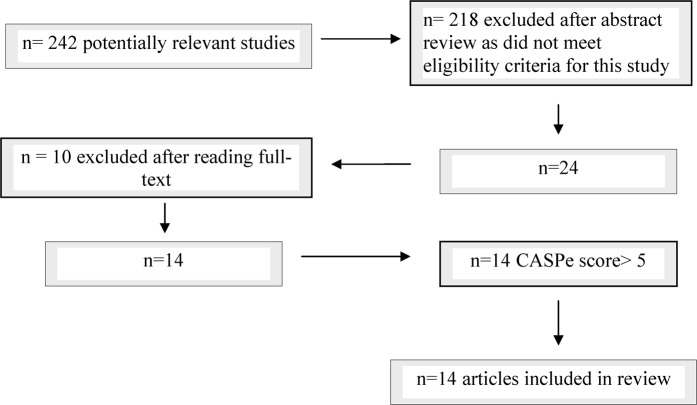
Flow-chart displaying selection of studies.

The initial search strategy retrieved 242 articles, which were reduced to 24 relevant to this review. These 24 articles were reviewed in full-text and 10 were excluded for not achieving the necessary criteria (Fig. 1).

### Methodological quality

Methodological quality as scored on the CASPe can be found in Table 1. There were no irresolvable disagreements between authors. All 14 studies scored greater than five. This CASPe tool has not been an elimination criterion. The studies included in this review share common threats to validity as most studies score negatively in the same areas. Frequently, a detailed description of the sample was absent and all studies failed to score for blinding or for the calculation of likelihood ratios.

**Table 1:** CASPe list for methodological quality assessment of studies

	Plamondon *et al.* (2007)^30^	Jasiewicz *et al.* (2007)^6^	Bourke *et al.* (2008)^33^	O’Donovan *et al.* (2007)^36^	Picerno *et al.* (2008)^11^	Martin-Schepers *et al.* (2010)^9^	Wong and Wong (2008)^8^	Zhou *et al.* 2008^32^	Zhou and Huosheng (2007)^13^	Musić *et al.* (2008)^12^	Roetenberg *et al.* (2007)^7^	Goodvin *et al.* (2006)^5^	Zhou and Hu (2010)^10^	Lee *et al.* (2010)^38^
Gold Standard	Y	Y	Y	Y	Y	Y	Y	Y	Y	Y	Y	Y	Y	Y
Description of the sample	Y	Y	Y	Y	N	N	N	N	N	N	N	N	N	N
Description of the experiment	Y	Y	Y	Y	Y	Y	Y	Y	Y	Y	Y	Y	Y	Y
Evaluation blinded	N	N	N	N	N	N	N	N	N	N	N	N	N	N
Neutrality in the results	Y	Y	Y	Y	Y	Y	Y	Y	Y	Y	Y	Y	Y	Y
Likelihood ratios	N	N	N	N	N	N	N	N	N	N	N	N	N	N
Accuracy of the results	Y	Y	Y	Y	Y	Y	Y	Y	Y	Y	Y	Y	Y	Y
Reproducibility of the test	Y	Y	Y	Y	Y	Y	N	N	N	N	N	N	N	N
Validity of the test	Y	Y	Y	N	Y	N	Y	Y	Y	N	N	N	N	N
Influence of the results	Y	Y	Y	Y	Y	Y	Y	Y	Y	Y	Y	Y	Y	Y
Total score	8	8	8	7	7	6	6	6	6	5	5	5	5	5

**Note:** Y = Yes N = No.

### Validity

A summary of the studies comparing motion analysis systems can be found in Table 2. Seven studies report correlation coefficients, six studies reported a coefficient of multiple correlation (CMC) values^8^,^11^,^13^,^30^,^32^,^33^ and three of these were focused on the measurement of the trunk (e.g. pelvis, lumbar and/or thoracic).^8^,^30^,^33^ One study focused on the lower limb (e.g. hip, knee and ankle).^11^ The remaining two studies reporting CMC values measured the upper limb, including the shoulder, wrist and elbow.^13^,^32^ In the measurement of the trunk, CMC values ranged from 0.829 for rotation of the pelvis^8^ to 0.998 for global pelvis angles rotation.^30^ Importantly, high CMC values were maintained across a wide range of tasks suggesting a good level of consistency for trunk motion measurement regardless of the gold standard used for comparison. CMC values for upper limb movements were excellent^32^ (especially in the elbow), as they were for the majority of lower limb kinematics investigated.

**Table 2:** Studies comparing inertial sensors with a video-based optoelectronic motion analysis system

Study	Description of study	Body area	Type of sensor/portability = size	Accuracy of the sensor				Gold standard	Validity				Participants
					Yaw	Pitch	Roll			Yaw	Pitch	Roll	
Plamondon *et al.* (2007)	The purpose of this study was to evaluate a hybrid system for the 3D measurement of trunk posture in motion.	T (TT, P)	Microstrain 3DM-G, Burlington weight 40 g. 64×64×25 mm	Global angles: P	2.0±0.5	0.5±0.2°	0.7±0.2°	Optoelectronic system (Optotrak 3020, Northern Digital Inc., Waterloo, Ont,, Canada)	Global angles: P (CMC)	0.998	0.974	0.975	*n* = 6 (6 male)Age (32±12 years)
				Global angles: TT	1.9±0.6	0.8±0.2°	0.7±0.1°		Global angles: TT (CMC)	0.988	0.993	0.971	
				Relative angles: P/TT	2.2±0.4	1.1±0.4°	1.6±0.8°		Relative angles: P/TT (CMC)	0.657	0.987	0.953	
Jasiewicz *et al.* (2007)^6^	The aim of this study was to determine the accuracy of new generation sensorsof wireless orientation.	T (CT)	Inertial Cube 3 sensor (Intersense, Bedford, MA, USA)/26.2×39.2×14.8 mm	Head mounted sensors	2.3±0.9	2.1±1.1°	2.5±0.9°	The 3-Space Fastrak (Polhemus, Colchester, VT, USA)	Head mounted sensors (cross-correlation)	0.97	0.98	0.97	*n* = 10 (mean age 33.4±9.9 SD, range 20–51 years)
				C7/Trunk mounted sensors	0.9±0.5	1.2±0.5°	0.7±0.7°		C7/Trunk mounted sensors (cross-correlation)	0.98	0.98	0.99	
Bourke *et al.* (2008)^33^	This study investigates distinguishing falls from normal activities of daily living by thresholding of the vertical velocity of the trunk.	T	ADXRS300 (Gyro) and ADXL210E (accel)/12×12×5 mm	RMS (M±SD): STSI = 0.09±0.05; Kneeling = 0.102±0.04; Object picking = 0.95±0.03; Lying on floor = 0.15±0.05; W = 0.08±0.03; Coughing = 0.06±0.02; Forward fall/knee FLX = 0.13±0.03; Side-fall right/Knee FLX = 0.15±0.09; Backward fall = 0.11±0.05				Optical motion capture system (6 cameras)	CMC (M±SD): STSI = 0.98±0.02; Kneeling = 0.96±0.03; Object kicking = 0.96±0.02; Lying on floor = 0.96±0.03; W = 0.89±0.07; Coughing = 0.73±0.29; Forward fall/knee FX = 0.98±0.01; Side-fall right/Knee FX = 0.98±0.02; Backward fall = 0.98±0.98				*n* = 5 (5 male)Age (25.6±1.9 years)
O’Donovan *et al.* (2007)^36^	The technique presented in this paper is concerned with ankle joint angles measurement.	LL (ankle)	ADXL210E (accel) ADXRS150 (Gyro) HMC2003 (mag) 60×40×24 mm	Angular errors in the measurement	3.33°	0.49°	–	Optoelectronic system (Evart 3D)					*n* = 2 (2 males)Age (25 and 23 years)
Picerno *et al.* (2008)^11^	This paper describes an anatomical calibration technique for three wearable inertial and magnetic sensing modules using palpable anatomical landmarks.	LL (hip, knee, ankle)	MTx (Xsens Technologies, The Netherlands)/weights 30 g. 38×53×21 mm	Hip absolute value (M±SD).	6.7±6.1	1.8±0.7°	3±2.2°	Optoelectronic system (Vicon Mx cameras, Oxford Metrics, UK)	The correlation coefficient for the FLX/EXT was equal to 1 for all the joints whereas the ÄRoM was less than 0.5°. The lowest *R* was the knee IER, and it was equal to 0.942				*n* = 1
				Knee absolute value (M±SD)	6.3±3.9	1.9±0.7°	4.6±1.1°						
				Ankle absolute value (M±SD)	8.3±1.6	1.3±0.9°	5.7±1.5°						
Martin-Schepers *et al.* (2010)^9^	This study proposes and evaluates an alternative algorithm for relative position and orientation. A complementary Kalman filter structure was presented.	TT, UL, LL	MTx (Xsens Technologies, The Netherlands)/weights 30 g. 38×53×21 mm	Orientation error: TT	4.3±0.3	4.5±0.7°	–	Optoelectronic system(Vicon, Oxford Metrics, UK)					*n* = 5
				Orientation error: UL	–	–	2.8±0.7°						
				Orientation error: LL	–	3.6±0.9°	–						
Wong and Wong (2008)^8^	The aim of this study was to introduce accelerometers and gyroscopes to detect posture in the sagittal and coronal planes.	TT (TT, LT, P)	KXM52-Tri-axis Kionix (Aceel) and Epson gyroscopes (Gyros)/22×9.20×9.12 mm, Weights 6 g	Peak value TT (degrees±SD)	–	22.8±11.1	3.8±1.5	Optoelectronic system (Vicon 370, Oxford Metrics, UK)	Correlation coefficient TT±SD	–	0.983±0.014	0.829±0.308	*n* = 5 (4 female and 5 male, age: 25.2±4.8 years, weight: 50.5±7.2 kg, height: 1.7±0.09 m)
				Peak value LT (degrees±SD)	–	24.7±7.0	6.2±2.2						
									Correlation coefficient LT±SD	–	0.981±0.014	0.984±0.015	
				RMS angular velocity (deg s^−1^±SD)	–	6.3±3.0	4.5±1.3						
Zhou *et al.* (2008)^32^	This paper presents a new human motion tracking system that is placed near the wrist and elbow joints.	Upper limb (shoulder, elbow, wrist)	MT9B (Xsens Technologies, The Netherlands)/weights 38 g. 39×54×28 mm	RMS elbow angles (degrees)	4.83	2.41	–	Optical motion tracker (CODA, Charnwood, UK)	Correlation coefficients in elbow	0.94	0.98	–	*n* = 4 (age range: 20–40 years)
Zhou and Huosheng (2007)^13^	A novel motion tracking prototype will be developed on the basis of the previously designed motion detector.	Upper limb (shoulder, elbow, wrist)	MTx (Xsens Technologies, The Netherlands)/weights 30 g. 38×53×21 mm	Arm position RMS (m)	–	0.004	0.005	Optical motion tracker (CODA, Charnwood, UK)	Correlation coefficients in arm	–	0.97	0.97	*n* = 4 (age range: 27–40 years)
Musić *et al.* (2008)^12^	Model validation was performed on simulated data and on measurements acquired with the Optotrak optical motion analysis system.	T, LL		Average self-selected STSI speed (Shank)	–	3.6°	–	Optotrak 3010 optical motion capture system (Northern Digital Inc., Waterloo, Ont., Canada),					*n* = 1
				Average self-selected STSI speed (Thigh)	–	5.2°	–						
				Average self-selected STSI speed (HAT)	–	5.8°	–						
Roetenberg *et al.* (2007)^7^	The objective of this study is to design and evaluate a new system for ambulatory measurements of position and orientation on the body.	T (TT), UL	MTx (Xsens Technologies, The Netherlands)/weights 30 g. 38×53×21 mm	Orientation error: TT	2.6±0.5	2.4±0.5°	2.6±0.5°	Optoelectronic system (Vicon 460, Oxford Metrics, UK)					*n* = 1
				Position error (mm): TT	4.9±1.0	4.8±1.1	5.0±0.9°						
				Orientation error: UL	–	2.4±0.5°	2.3±0.5°						
Goodvin *et al.* (2006)^5^	They propose a new method for accurately measuring the real-time orientation and position of the spine in a portable, non-invasive, and clinically meaningful manner.	T (CT, TT, LT)	MT9B (Xsens Technologies, The Netherlands)/weights 38 g. 39×54×28 mm	Cervical average deviation	0.2°	0.42°	0.1°	Optoelectronic system (Vicon 460, Oxford Metrics, UK)					*n* = 5
				Torso average deviation	0.23°	0.06°	0.03°						
				Hip average deviation	1.35°	0.33°	3.1°						
			
			
Zhou and Hu (2010)^10^	This paper presents the effects of changes in error reduction by using Kalman filtering.	Upper limb (shoulder, elbow, wrist)	MTx (Xsens sTechnologies, The Netherlands)/weights 30 g. 38×53×21 mm	Statistical error before Kalman filter	–	14.62°	14.02°	Optical motion tracker (CODA, Charnwood, UK)					*n* = 4
				Statistical error after Kalman filter	–	2.13°	2.01°						
Lee *et al.* (2010)^38^	In this study they present sensor nodes (accel) with a goniometer probe.	UL	Freescale MMA7261QT (accel)/6×6×1.45 mm	A linear increasing trend from 0±2.5° at a mean angular speed of 10° s^−1^ to 3.5±7° at 80° s^−1^.				Goniometer probe (PS-2137 from PASCO)					*n* = 1

**Note:** ADL, activities of daily live; CMC, coefficient of multiple correlation; M, mean; SD, standard deviation; STSI, sit-to-stand; FX, flexion-extension; FLX, flexion; EXT, extension; ABD, abduction; ADD, adduction; IER, internal–external rotation; PT, protraction; RT, retraction; MLR, medio-lateral rotation; APT, anterior–posterior tilting; RMS, root mean square; ACRL, angular coefficient of the regression line; IQR, inter-quartile ranges; P, pelvis; LB, lateral bending; R, rotation; TT, thoracic trunk; UL, upper limb; LL, lower limb; G, gait; CT, cervical trunk; LT, lumbar trunk; T, trunk.

### Accuracy of the sensor

Thirteen studies reported an error measurement in degrees, with four of these studies reporting the root mean square error. Eight studies reported error measurements for analysis of trunk motion, including the cervical region. Greatest errors were reported by Wong and Wong,^8^ which ranged (in absolute values) from 22.8 to 24.7°, for thoracic and lumbar regions, respectively. In contrast, Jasiewicz *et al.*^6^ reported trunk monitoring errors of less than 0.7° for the coronal plane spine, errors of less than 1.2° for the saggital plane measurement and errors of less than 0.9° for rotation of cervical spine. Greater errors were reported by Martin-Schepers *et al.*^9^ for thoracic motion (ranging from 4.3 to 4.5°). Roetenber *et al.*^7^ found error values in the thoracic trunk to range from 2.4 to 2.6°. Plamondon *et al.*^30^ and Goodvin *et al.*^5^ gave angular error results in average values. In this case, the values in the thoracic trunk were in the range of 0.03–0.7° for the lateral bendings. The average error values were always less than 2.2°.

It appears that errors associated with upper limb movement are more consistent, with a range reported from 2.3° (Ref. 7) to 4.83°.^32^ The study of Zhou and Hu^10^ confirmed the effectiveness of the Kalman filter, providing results before and after the filter of 14.62–2.13°. However, the lower limb appears relatively inconsistent with errors ranging from 0.49° (Ref. 36) to 8.3°.^11^

### Portability

The sensor size ranged from 64×64×25 mm for the larger sensor to 12×12×5 mm for the smaller sensor. All sensors are portable, either wireless or with single wire attachment, however the sensor size is an important consideration depending on the anatomical region to be investigated.

## Discussion

In this review, 14 studies were identified, which compared directly inertial sensors to any kind of gold standard for human motion analysis (e.g. electrogoniometry, optoelectronic systems, electromagnetic systems, etc.). This review provides the first synthesis of the studies relating to the validity, reliability and accuracy of inertial sensors compared to accepted technology; this gold standard has to be a tool for measuring human movement. It appears that inertial sensors can be applied to many body regions accurately and reliably. The degree of accuracy and reliability displayed suggests that it can be used to measure repeatedly specific motions in varying contexts. The actual degree of reliability is site specific but it is evident that inertial sensors provide a viable option for motion analysis.

The diversity in the reported studies precludes a simple synthesis of results. A systematic comprehensive analysis of the results was not considered to be appropriate given the diversity among a fairly small number of studies, the varied sample and the heterogeneity of movements studied, the marked variability in the quality of the data, differing methods and statistical analysis and the heterogeneity of results. Most studies were of poor methodological quality with studies related to the development and calibration of the sensors less important to the authors. Under these circumstances, the review comprised a pseudo quantitative analysis of the research available.

Whether inertial sensor data are reliable enough remains a question that can be answered in the context of the proposed use, with the degree of acceptable measurement variation relating directly to the intended application. Clearly, it is beyond the scope of this review to specify the acceptable limits of reliability for all possible clinical applications of inertial sensors. Following McGinley *et al.*’s^40^ research, we accepted that in most common clinical situations, an error of 2° or less is considered acceptable, as such errors are probably too small to require explicit consideration during data interpretation. Errors of between 2 and 5° are also likely to be regarded as reasonable but may require consideration in data interpretation. We suggest that errors in excess of 5° should raise concern and may be large enough to mislead clinical interpretation. Data from the studies reporting errors revealed that the majority of studies show errors that fall between 2 and 5°. Thoracic and lumbar trunk clearly showed the highest error,^8^ although it is noteworthy that some studies reported lower error of 2° for the same variable, suggesting that the lower error is currently achievable.^5^,^7^,^9^,^30^

The benefits of such a system for the clinician and researcher lie in its inherent portability, accuracy and reliability in the context of proposed use. The sensors are either connected to a personal computer, data logger or may be operated wirelessly providing a wide variety of applications. This freedom enables the system to be used in any environment. Furthermore, these systems can be operated over a range of sampling frequencies enabling tasks of long duration to be studied, such as sitting at a desk at work;^8^ very rapid tasks, such as the golf swing can also be studied. Algorithms can be created to provide real-time feedback to the user providing an instant tool to observe and correct motion.^10^

Common threats to validity are evident throughout the studies and some important aspects should be considered. Appropriate sample composition and inclusion/exclusion criteria should ensure that the range of characteristics of interest in a clinical target population is most likely to be present in a sample, and that the findings can be generalized. It is evident that the studies reviewed failed to describe adequately the baseline characteristics, limiting the reader’s understanding of the threat this poses to external validity. The potential influence of the assessor characteristics on the reliability of inertial tracking devices data received limited focus within the studies in this review. Due to the complexity of understanding three-dimensional kinematics and the necessity for the development of automated algorithms, a level of expertise and experience may be important in identifying and removing sources of error. Furthermore, models often require the use of landmark-specific markers, the placement of which may influence accuracy and reliability.

Although the majority of studies described the use of standardized protocols, wide variation was apparent in the duration between measurement sessions. The selection of an optimal interval in repeated measures requires consideration of both practical and theoretical issues. In principle, intervals should be designed to minimize fatigue and biological variation associated with repetitive human motion.^41^ Artificially-short intervals are often most feasible, yet the presence of visible marker residue could ‘unblind’ a repeat assessment and may influence results.

Blinding of assessors to prior measurements is typical practice within repeatability studies. Although the potential for assessor bias is less apparent with instrumented measures, it remains a potential factor in some of the studies reviewed. It is particularly important to be blinded prior to measurements in comparative studies.

A fundamental question in the reliability of inertial tracking devices is whether the measures are reliable enough for clinical decision-making. Although indices, such as the CMCs and other correlation coefficients were commonly reported, it is now well-recognized that, in isolation, correlation indices do not tell us whether the measures are reliable. It provides a measure of similarity between the systems but is not a measure of the difference. To make a proper assessment, reliability measures of both are required. This enables the degree to which a system can resolve measurements of interest to be determined, and should be presented in the data.^42^ This is a significant limitation of much of the existing literature, with about half of the papers only reporting errors in absolute terms. It is recommended that future studies investigating reliability of inertial sensors include measures of absolute error.

The prevalence of reports using the CMC warrants particular attention as the calculation method of the CMC is influenced markedly by the joint range of motion.^5^,^6^,^30^,^32^ In those movements which require the contribution of a large number of joints, such as walking, the CMC value is lower.^33^ By contrast, in studies utilizing a more specific joint, the CMC is much higher.^8^,^6^,^13^,^30^ This is complicated when studying a number of joints and even more complex when studying a global movement, such as gait. Therefore, it is acknowledged that the greater the number of joints involved in the study, the lower the ensuing reliability.

This review is limited to those articles identified by the search strategies, and study quality was reviewed only by the criterion tool, CASPe. Future studies of the reliability of inertial tracking devices require careful consideration of optimal design to enhance the generalizability of the findings. If the intention is to apply the reliability estimates to clinical populations, then careful attention is necessary to recruit and describe samples, which are representative of the clinical populations of interest. Protocols should consider carefully what standardized measurement interval is most appropriate and minimize predictable sources of assessor bias. Appropriate statistical strategies should include reliability estimates and errors in units of degrees to enhance interpretation. The refinement and optimization of test protocols will help enable the minimization of errors.

## Conclusion

This review concludes that inertial sensors can offer an accurate and reliable method to study human motion, but the degree of accuracy and reliability is site and task specific. They are able to measure differing body regions and overcome the problem of line-of-sight or metallic disturbance associated with other methods. They offer a tool, which has the potential to span many applications in many environments outside of a laboratory and therefore, they warrant further development to continue to improve their systems and their application for human motion analysis.
